# Developing a music-based digital therapeutic to help manage the neuropsychiatric symptoms of dementia

**DOI:** 10.3389/fdgth.2023.1064115

**Published:** 2023-01-20

**Authors:** Frank A. Russo, Adiel Mallik, Zoe Thomson, Alexander de Raadt St. James, Kate Dupuis, Dan Cohen

**Affiliations:** ^1^ Department of Psychology, Toronto Metropolitan University, Toronto, ON, Canada; ^2^ KITE, Toronto Rehabilitation Institute, University Health Network, Toronto, ON, Canada; ^3^ LUCID Inc., Toronto, ON, Canada; ^4^ Center for Elder Research, Sheridan College, Oakville, ON, Canada; ^5^ Right to Music, New York, NY, United States

**Keywords:** digital therapeutics, dementia, neuropsychiatric symptoms, anxiety, agitation, music, artificial intelligence

## Abstract

The greying of the world is leading to a rapid acceleration in both the healthcare costs and caregiver burden that are associated with dementia. There is an urgent need to develop new, easily scalable modalities of support. This perspective paper presents the theoretical background, rationale, and development plans for a music-based digital therapeutic to manage the neuropsychiatric symptoms of dementia, particularly agitation and anxiety. We begin by presenting the findings of a survey we conducted with key opinion leaders. The findings highlight the value of a music-based digital therapeutic for treating neuropsychiatric symptoms, particularly agitation and anxiety. We then consider the neural substrates of these neuropsychiatric symptoms before going on to evaluate randomized control trials on the efficacy of music-based interventions in their treatment. Finally, we present our development plans for the adaptation of an existing music-based digital therapeutic that was previously shown to be efficacious in the treatment of adult anxiety symptoms.

## Introduction

According to the World Health Organization (2022), there are 55 million people living with dementia worldwide with 10 million new cases annually. The same report estimates the global cost of dementia at 1.3 trillion USD (1). These costs are expected to surpass 2.8 trillion USD by 2030 as the number of people living with dementia rises. Approximately half of the global cost of dementia is attributable to the informal care provided by family members and friends who commonly shoulder tremendous physical, emotional and financial pressures (2). A sizeable minority of people living with dementia in industrialized societies will eventually be placed in long-term care (nursing or assisted care) homes. The proportion of total costs incurred in these homes that can be attributed to dementia has been estimated at 64% (3). Even before the COVID-19 pandemic, the professionals in these homes were chronically overloaded leading them to experience high levels of caregiver burden, and in some cases, moral injury, which has been defined as the perpetration, failure to prevent, or observation of morally-transgressive acts (4).

From the perspective of people living with dementia, their caregivers and the broader healthcare system, there is an urgent need to develop new, easily scalable modalities of support. Digital therapeutics (DTx) represent one such modality being considered. The focus of development in DTx for dementia has been cognitive stimulation (5), typically in the form of reminiscence therapy (6) or brainwave entrainment (7). The objective of such therapeutics is to directly slow the rate of cognitive decline (8). While we believe that interventions that directly target cognitive outcomes are clinically important, we also believe there is an urgent need to target non-cognitive outcomes. These outcomes have been less well studied in the context of DTx but they have the potential to contribute to patient and caregiver wellbeing, while lowering the costs of care (9).

Our team is currently undertaking the development of a music-based DTx that builds on core AI that we originally developed to mitigate anxiety (https://www.lucidtherapeutics.com). While the anecdotal evidence for the power of music in dementia abounds, the evidence base is still in its early days and tends to be focused on cognitive outcomes. To better understand the potential impact of a music-based DTx on cognitive and non-cognitive outcomes we started our development path by surveying key opinion leaders.

### Survey of key opinion leaders

In early 2022, our team undertook a qualitative study with key opinion leaders to gauge the potential value of developing a music-based DTx for dementia. In addition to defining the value proposition, we were interested in specific outcomes that were judged to be feasible, inclusive of cognitive and non-cognitive outcomes. Participants included 7 payers and 12 health-care practitioners specializing in geriatric and dementia care. Payers included medical directors (*n* = 5), pharmacy directors (*n* = 1), and an innovation officer (*n* = 1) associated with health plans that are based in the United States. All payers had experience with the evaluation of DTx for coverage and reimbursement. Health-care practitioners (HCPs) included neurologists (*n* = 5), geriatricians (*n* = 4), and psychiatrists (*n* = 3), all of whom had significant experience in treating Alzheimer's disease (AD) and other forms of dementia (50 patients or more in the last 3 months). Most of the HCPs surveyed had experience with use of DTx in treatment (75%), and about half had some experience in recommending music therapy for patients (58.3%). Both payers and HCPs expressed the view that there was a strong clinical case for a therapy/intervention that would target non-cognitive aspects of dementia. In particular, they identified the *neuropsychiatric symptoms* (10) as being a non-cognitive target outcome that might be well addressed by a music-based DTx. Neuropsychiatric symptoms are extremely common in dementia, affecting as much as 97% of patients (11) and have been associated with reduced quality of life (12), as well as the progression of cognitive decline (13, 14).

### Neuropsychiatric symptoms: prevalence, caregiver challenge, and neural substrates

In descending order of frequency, neuropsychiatric symptoms of dementia include apathy, depression, agitation, psychosis, and sleep disturbances (15). Agitation is especially frequent (80%) in residents of long-term care homes and in those who are in moderate to severe stages of the disease (16) but can also affect many individuals (60%) with mild dementia or mild cognitive impairment (MCI) (17). According to the key opinion leaders we surveyed, agitation is the most challenging symptom with respect to patient management. This perspective is consistent with prior surveys conducted with caregivers. One study of American caregivers found agitation to be more distressing than apathy or depression (18). The same conclusion was reached in a study of caregivers conducted in Japan (19). The Japanese study also found that agitation was more likely to contribute to caregiver burnout than other neuropsychiatric symptoms.

In addition to the challenge that agitation presents for caregivers it has also been associated with the progression of cognitive decline in patients (13, 14). From a biopsychosocial model of cognitive aging (20), this association may be attributable to neurotoxic factors that manifest due to stress arising from frequent bouts of agitation (see (21). In the case of patients living in long-term care homes, the association may also be due to side effects of the antipsychotic medications that are commonly prescribed to treat agitation. A meta-regression involving data from ten studies found a strong linear correlation between antipsychotic treatment duration and change in cognition, with greater declines under antipsychotic treatment compared to placebo (22).

Risperidone, a commonly prescribed second-generation antipsychotic with the strongest evidence base for treating agitation and anxiety appears to have no adverse effects on cognition when prescribed as indicated for short-term use (23–25). However, side effects of risperidone include an elevated risk of ischemic stroke and transient ischemic attacks (26), which elevate mortality risk. Studies of other commonly prescribed second-generation antipsychotics, such as olanzapine, have shown some level of risk for cognition, especially in the case of participants with lower cognitive functioning at baseline (27). Haloperidol, a first-generation antipsychotic that continues to be prescribed for agitation is less efficacious than risperidone (28), has potent sedative effects, and was determined to be the riskiest of all pharmacological interventions with respect to mortality (29). In summary, while chronic agitation may hasten the progression of cognitive decline, the existing pharmacological approaches have limited efficacy and can carry significant risks to physical health (30) and cognitive health (22). The healthcare practitioners we surveyed were particularly interested in the development of DTx that would serve as complementary or low-risk alternatives to pharmacological treatment in the management of agitation and anxiety.

Some researchers have characterized agitation as the external manifestation of anxiety (31, 32). Up to 80% of people living with mild-to-moderate dementia experience anxiety (15). Anxiety may even be a risk factor for developing dementia; the risk of conversion to dementia nearly doubles when anxiety symptoms are present in people living with mild cognitive impairment (33). While agitation is more of an external behavior that is readily observable, anxiety is an internal state that can be hidden from plain view. It has been conceptualized as consisting of cognitive (i.e., worry about future threats) and somatic (i.e., bodily tension) components (34). Anxiety tends to be more common in the early stages of the “dementia journey”, while agitation is more common in later stages (35). Although the anxiety does not appear to be causally related to later agitation as has often been proposed (32), there is a clear association between the two constructs across stages of disease (36).

The ‘Uncertainty and Anticipation Model of Anxiety’ (UAMA) posits that anxiety is a set of expected emotional, cognitive, and behavioural responses to the uncertainty of potential future threats, often coupled with fear (37, 38). The UAMA model proposes that activity in the frontal cortex (dorsomedial prefrontal and orbitofrontal) is responsible for generating probabilistic estimates of future events and expected costs (37). The model also proposes that the amygdala plays a central role in the transmission and interpretation of anxiety and fear. In addition to afferents from the frontal cortex, the amygdala is known to receive afferents from the thalamus, periaqueductal gray, and entorhinal cortex (38–40). People living with a diagnosis of dementia tend to experience a great deal of uncertainty because of the unknown of how their illness will progress and not knowing what threats may await them (41). Neural degradation in frontal areas supporting working memory may further predispose individuals living with dementia to experience anxiety.

In the case of AD, the most common form of dementia, anxiety is associated with damage to subcortical regions which includes atrophy in the amygdala (38, 42) and the entorhinal cortex (43). Cases of more severe anxiety are associated with hyperfusion of the anterior cingulate cortex, decreased grey matter volume in the right precuneus, inferior parietal, left parahippocampal, posterior cingulate gyrus, left insula, and bilateral putamen lobes (37, 38) and hypometabolism in the bilateral entorhinal, anterior hippocampus, left superior temporal and insula regions (38, 44). Positron emission tomography (PET) studies indicate that individuals living with AD and comorbid anxiety possess higher amyloid deposits than those without in the precuneus-posterior cingulate, frontal, parietal, and anterior cingulate cortex (45).

**Figure 1:**
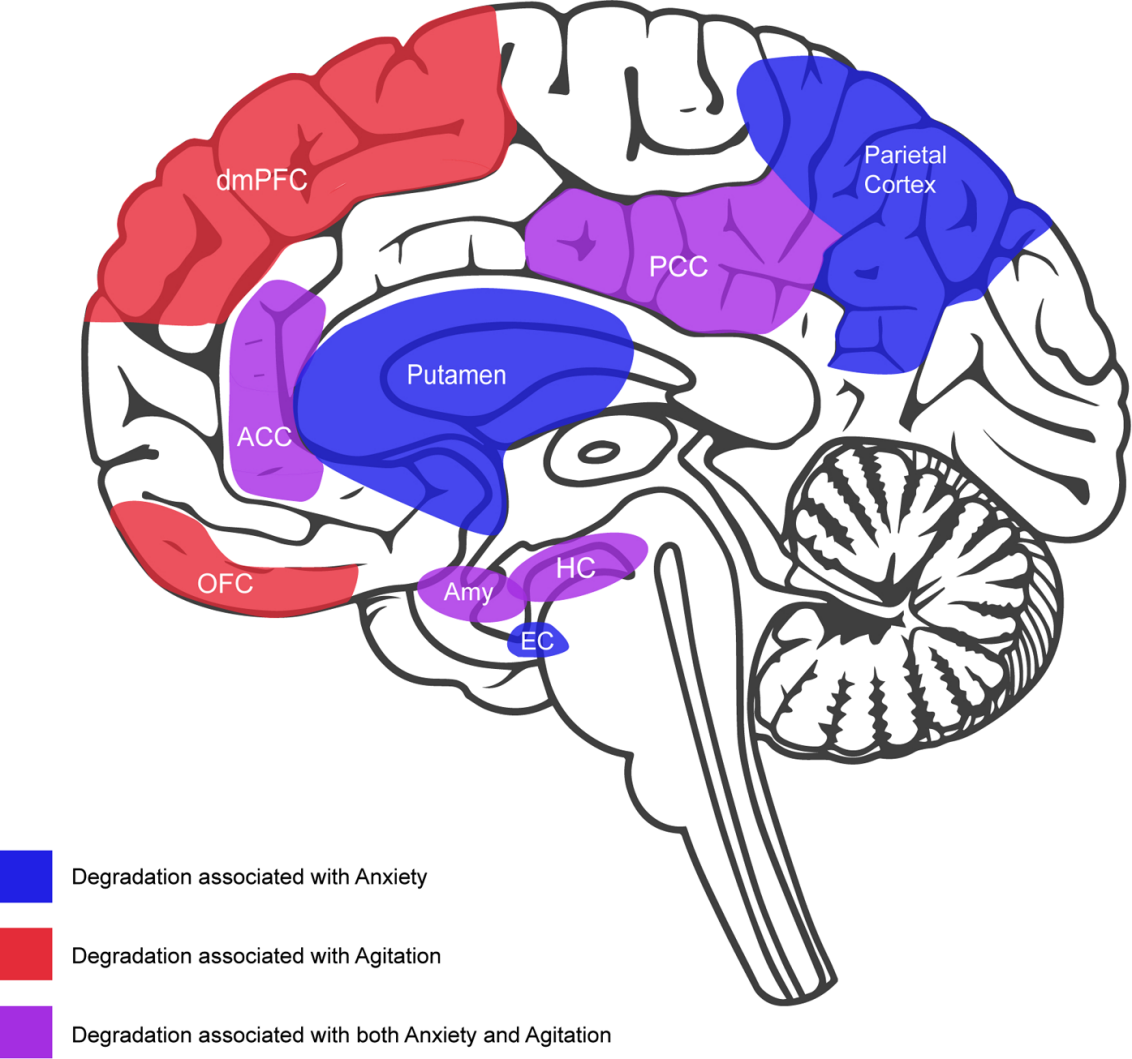
Midsaggital view of brain featuring neural degradation that has been associated with anxiety and agitation in dementia: anxiety (blue) = putamen, parietal cortex, and entorhinal cortex (EC); agitation (red) = orbitorfrontal cortex (OFC); dorsolateral prefrontal cortex (dmPFC); anxiety and agitation (purple) = anterior cingulate cortex (ACC), posterior cingulate cortex (PCC), amygdala (Amy), hippocampus (HC), and insula (not depicted in this view).

As shown in Figure 1, individuals living with AD that present with agitation show severe dysfunction in many of the same brain regions that are implicated in anxiety, including the amygdala, hippocampus, anterior cingulate, posterior cingulate, and insula (46). This pattern of dysfunction agrees with the pattern of disease progression wherein the propensity for anxiety is higher in earlier stages while the propensity for agitation is higher in later stages once more severe brain dysfunction, particularly dysfunction in frontal areas, has occurred (35). Although the type and onset of neural degradation that occurs in the frontal lobes will vary by type of dementia (47), at later stages of the disease, these degradations may uniformly result in the failure to downregulate autonomic arousal in response to uncertainty (37). Taken together, the available evidence suggests that anxiety and agitation are independent but related constructs whose propensity will be influenced as a function of neuropsychiatric disease progression. It stands to reason that the two types of neuropsychiatric symptoms may benefit from similar types of intervention. In recent years, music has emerged as a particularly important intervention for neuropsychiatric symptoms, especially with respect to anxiety and agitation. When used in this context, music may be regarded as a fundamental technology that can be personalized and systematically leveraged to downregulate autonomic arousal arising from uncertainty.

### Are music-based interventions indicated for the treatment of anxiety and agitation symptoms related to dementia?

Qualitative research that has examined the impact of music-based interventions on people living with dementia has revealed significant improvements in quality of life in patients and caregivers. Notable benefits in patients include increased social engagement and reductions in anxiety and agitation (48–54). To further understand these benefits and for whom they may accrue, we conducted a literature search focusing on randomized controlled trials (RCTs) that investigated the use of music-based treatments on anxiety or agitation symptoms in participants with MCI and/or dementia. Of the 15 RCTs found, three RCTs were excluded due to low fidelity (55) and no personalization of music/music therapy (56, 57). Of the remaining 13 RCTs, 12 of them reported a significant reduction in anxiety and/or agitation in the music/music therapy treatment arm (Table 1). The most parsimonious interpretation regarding the mechanism of action is a downregulation of autonomic arousal, owing to a shift in balance from sympathetic to parasympathetic activity over the course of music listening (70). Overall, the effect sizes (Cohen's D) trended larger in studies that recruited participants with mild to moderate dementia. It is also notable that the one study that failed to find a significant reduction in anxiety and agitation was limited to participants with severe dementia (64). Thus, based on the available evidence we may conclude that agitation and anxiety symptoms are well-indicated for music-based treatment, especially in participants with mild to moderate dementia.

**Table 1:** Overview of dementia and music therapy randomized control trials.

Study	Conditions	Music Intervention	Dementia Stage	Outcome Measures	Main Findings	Effect Size (Cohen's D)
(58)	Music vs. Passive Control (standard care)	Songs familiar to participants were used. 30 min twice a week for 6 weeks.	Diagnosed with dementia but able to follow simple instructions (assume mild to moderate)	Rate of Anxiety in Dementia Scale (RAID), Cohen-Mansfield Agitation Inventory (CMAI)	Decreased anxiety; no difference in agitation	0.90
(59)	Music Therapy (MT) vs. Active Control (reading)	Individualized MT method used. Music chosen according to patients’ personal tastes.	Mild to moderate AD type dementia	Hamilton Anxiety Scale, Geriatric Depression Scale (GDS)	Decreased anxiety; decreased depression.	1.23–2.48 for anxiety.
(60)	Music vs. Active Control (multi-sensory stimulation)	Music therapist selected music for each patient considering patients’ musical tastes	Severe dementia	CMAI, RAID, Cornell Scale for Depression in Dementia (CSDD)	Both groups had a decrease in anxiety; no effect for either group on agitation.	N/A
(61)	Music therapy (MT) vs. Active Control (recreation activities)	Music therapist selected music to incite pleasant memories and reduce agitation based on musical parameters (slow tempo etc.) (not personalized)	DSM-IV diagnosis of dementia with high level of behavioural problems indicated by Cohen-Mansfield Agitation Inventory >44; broad range but predominantly moderate to severe	CMAI	Both interventions showed a decrease in agitation; but no statistical difference between the treatment and control groups	N/A
(62)	Music Intervention vs. Passive Control (standard care)	Popular music was selected from the time of patients’ youth. Not individually personalized.	Diagnosed with dementia DSM-IV and older than 65; broad range but predominantly moderate	C-CMAI	Reduction in agitated behaviour, physically aggressive behaviour	N/A
(63)	Music listening vs. Controls (singing vs. standard care)	Used familiar songs for music listening and singing groups not individually personalized to participants	Those unable to complete study measures were excluded (assume mild to moderate)	Mini-Mental State Exam (MMSE), Cornell-Brown Scale for Quality of Life (CBS)	Music listening and singing groups had reduction in behavioural symptoms including agitation	N/A
(64)	Music Therapy (MT) vs. Controls (individualized music listening vs. standard care)	MT was based on interactions (verbal/instrumental) between the patient and therapist. IML contained music selections made by music therapist based on interviews with patient's caregivers and the patient.	Moderate to severe	MMSE, Neuropsychiatric Inventory (NPI), CBS, CSDD	Overall behavioural assessment did not show significant differences between treatment and controls. Significant improvement over time in anxiety subscale for all groups. No effect on agitation.	N/A
(65)	Music Therapy (MT) vs. Passive Control (standard care)	MT was based on interactions (verbal/instrumental) between the patient and therapist. Well known songs were used in addition to improvisation.	Display at least two neuropsychiatric symptoms but no significant health problems (assume mild to moderate)	NPI-NH, dementia care mapping (DCM)	Caregivers reported an improvement in managing anxiety and other dementia symptoms. Increased observed well-being in MT group, reduced neuropsychiatric symptoms in MT group.	2.32–2.69
(66)	Music Therapy (MT) vs. Active Control (recreational activities)	Unknown level of personalization. Residents actively engage in music making/singing or listening to music that the music therapist plays or sings.	Nursing home residents with diagnosis of dementia (not enough information to infer stage)	NPI-Q	Agitation and other dementia behaviours were significantly reduced in MT group according to NPI-Q.	N/A
(67)	Music Therapy (MT) vs. Active Control (recreational activities)	MT was based on interactions (verbal/instrumental) between the patient and therapist.	Moderate to severe	NPI, MMSE	Agitation, anxiety, and other dementia behaviours/symptoms were significantly improved according to NPI total score.	0.53–1.8
(68)	Music Therapy (MT) vs. Active Control (recreational activities)	MT based on non-verbal model and on sound-music improvisation using musical instruments.	Severe	NPI, MMSE, Barthel Index (BI)	Agitations, delusions and apathy significantly improved in Music therapy group but not in control group.	0.63
(69)	Music Therapy (MT) vs. Passive Control (standard care)	Individual music therapy: vocal or instrumental improvising, singing, dancing, or listening to familiar or unknown songs	Moderate to severe	CMAI, ADRQL	Agitation decreased significantly during music therapy compared to control.	0.50

### Current standard of care with respect to therapeutic uses of music

Accumulating evidence demonstrates that people living with dementia enjoy music and may benefit from making music, interacting with music through movement, and passive listening to music (71, 72). It has been shown that people living with AD can retain memory for melodies and lyrics (73), and that when activated, these memories facilitate the retrieval of autobiographical memories (74). The mechanisms underlying these music and memory phenomena are not completely understood but appear to depend on the encoding of musical memories in structures and networks that are resilient to neural degeneration (75). It seems likely that these music and memory phenomena depend in part on dopaminergic activity in the ventral striatum triggered by auditory-reward network connectivity, which is well preserved in MCI but less so in AD (76).

Music and memory phenomena have sparked widespread interest in popular culture, particularly following the release of the 2014 documentary film *Alive Inside*. The film tells the story of MUSIC & MEMORY, a non-profit that facilitates the use of music players for people living with dementia in long-term care homes. In an often-cited highlight of the film, a long-term care home resident named Henry is jolted out of his catatonic state into a charming, articulate, and engaged lover of music. The film also chronicles the struggle that founder Dan Cohen had in convincing healthcare professionals and administrators about the value of music in care for people living with dementia. MUSIC & MEMORY program has since expanded and has been imitated with variations all over the world and validated in a number of large-scale trials (77, 78).

In community and long-term care homes, therapeutic music can be observed in various modalities from music listening to support mood and reminiscence (79), singing to promote health and social wellbeing (80), and music-making coordinated by a licensed music therapist (81). Regardless of the modality, a scoping review of the literature suggests that personalized music is systematically more effective than non-personalized music (82, 83). Personalization is likely important because familiar and preferred music leads to greater activation of dopaminergic and opioid pathways in the ventral striatum than nonfamiliar music (84, 85), and in the case of AD this reward activation is also associated with greater functional connectivity in corticocortical and corticocerebellar networks (86). Personalization is further supported by the fact that people with AD have dysfunctional dopaminergic (87) and opioid transmission (88) and stimulating production of endogenous opioids through music (85) or other means may have a beneficial impact on anxiety and agitation over the near-term and potentially slowing the progression of disease over the long-term (88).

Because personalization is so important to the effectiveness of music it stands to reason that a limiting factor in scalability of any effective music program will be the time and effort required to personalize music for a given individual. This may be especially challenging when the caregiver has limited experience with the person living with dementia and/or the individual has limited communication abilities. A licensed music therapist would be able to cultivate some level of personalization through careful interaction and observation with an individual. However, there are barriers to accessing music therapists, which limits the benefits that may be obtained from music engagement. A music-based DTx will help bridge the gap by offering the level of personalization required for optimal outcomes in the absence of a licensed music therapist.

The music based DTx is not intended to diminish or replace the benefit that trained music therapists may have in both group and individual music therapy sessions. It cannot augment or alter the benefit patients receive from the therapeutic “relationship” that results from co-engagement of both passive and active music interventions, whether those occur in the presence of music therapists or caregivers without this training. It may, however, increase the opportunities for caregivers of all types to advance the therapeutic relationship by coming to learn about their patients in new ways. Lastly, digital interventions that capture continuous physiological data reflecting patient experience with music (e.g., heart-rate variability, pupillometry) may empower caregivers with a level of insight about response to music that would not otherwise be possible, which may inform all manner of music-based interventions including traditional music therapy as well as multi-modal interventions involving music such as augmented reality applications.

To this end, the user interface of the DTx will be designed in a manner that is conducive to operation by either a caregiver in the community (e.g., family member) or a professional in a long-term care home (e.g., music or recreation therapist). There will be no expectations imposed regarding caregivers' level of experience with music, nor will there be any requirement of familiarity with the music preferences of the person they are caring for.

### Adaptation of a music-based DTx for managing anxiety

Our team has recently published an RCT study on the efficacy of a music-based DTx developed by LUCID (https://www.lucidtherapeutics.com) for the treatment of anxiety (89). The system incorporates an AI called Affective Music Recommendation System (AMRS) (90); based on the iso-principle from music therapy (90, 91), which is a form of personalization that is independent of experience or preference. This approach to mood regulation suggests that the mood regulating properties of music may be enhanced if the mood of the music approximates an individual's initial emotional state before it is changed to the target state (92). The iso principle has been indicated in prior research to be more effective than other musical sequences at reducing tension (93). To develop AMRS, it was necessary to begin by curating training data that labeled music with respect to the arousal and valence dimensions of the Russell's Circumplex model of emotion (94). In this model, arousal refers to the activation aspect of felt emotion, ranging from calm to excited, and valence refers to the hedonic aspect of felt emotion, ranging from pleasant to unpleasant.

In LUCID's existing DTx (VIBE), the participant is asked to input their current mood using a 2-dimensional grid representing arousal and valence dimensions. Based on this input and the user's target emotional state (e.g., calm), the machine learning algorithm within the application predicts the optimal sequence of tracks to produce mood induction in the listener from their current emotional state to the target state. This machine learning algorithm uses reinforcement learning techniques and is trained on real-world data correlating the quantitative features of musical excerpts and sequences alongside the emotional responses induced by them in listeners.

### Developing a music-based DTx for people living with dementia

Our existing DTx (VIBE) was designed to reduce anxiety. This objective was realized through the interaction of two AI systems: BioMIR (Biological Music Information Retrieval) and AMRS (as described above). The BioMIR system extracts insights about the emotional states that are likely to be evoked by pieces of music in people living with dementia. The AMRS considers the information from the BioMIR system to generate playlists that are personalized to each user. People living with dementia often have a diminished ability to attribute mental states to music, inclusive of emotion (95). For example, a musical excerpt that may calm a healthy person down may have a different effect on a person living with dementia. Therefore, to aid in the development of a new AI for affective music recommendation in this population (AMRS-D), we had to start by obtaining training data from older participants experiencing cognitive decline. To that end, we recently completed a training database study in collaboration with the Centre for Elder Research at Sheridan College with 32 participants living with MCI or early-stage dementia.

Participants were asked to listen to and make self-report judgments of valence, arousal, and absorption. Absorption was defined as the extent to which attentional resources were allocated to the music while listening (96, 97). It is our expectation that higher levels of musical absorption will lead to increased potency of a music-based intervention for mood regulation. While this hypothesis has not yet been validated with respect to state absorption in people living with dementia, it has been validated for trait absorption in a young adult population (see (98, 99)). These subjective judgments were collected alongside a variety of biometric measures which will allow us to use industry-standard machine learning methods to develop a fully closed-loop music recommendation system that can be driven by physiological data alone independent of user input (100). Going forward, the effectiveness of the DTx may be further enhanced by embedding beat stimulation in the theta range (90) with the expectation of increasing the extent of reduction in anxiety and agitation (89).

After the development of AMRS-D and the absorption module, we will begin an exploratory trial to assess the useability of the new system, including early indications of safety and efficacy. The exploratory trial will recruit participants living with dementia in the community by way of their caregivers. The DTx will be used by caregivers on a scheduled daily basis rather than in response to anxiety or agitation in the patient. The exploratory trial will provide an opportunity to solicit qualitative feedback that will lead to product refinement and pave the way for a future clinical proof-of-concept study. In this future proof-of-concept study, adherence and clinically relevant measures of efficacy will be tracked including the Neuropsychiatric Inventory Questionnaire (NPI-Q (101);, Behavioral Pathology in Alzheimer's Disease Rating Scale (BEHAVE-AD) (102), State-Trait Inventory of Cognitive and Somatic Anxiety (STICSA) (103) and the Cohen-Mansfield Agitation Inventory (104).

## Discussion

In this paper we have outlined the rationale for a new music-based DTx that LUCID is developing to support the neuropsychiatric symptoms of dementia. This development represents an expansion of our prior work in adult anxiety (89) to help support a related indication in the context of persons living with dementia (i.e., agitation). We have argued that a music based DTx will be effective in mitigating both anxiety and agitation in this population. We envision our music-based intervention as having efficacy at all stages of disease but with the focus of benefits on anxiety in the early stages, and in agitation in the later stages. The DTx will be proactive rather than reactive. Scheduled daily use of the system is projected to lead to improvements in patient and caregiver outcomes and reduced costs of care. The DTx will respect individual preference for music through a personalization module that will eventually be implementable in the absence of caregiver input. The available evidence suggests that patients will find intrinsic benefits in listening to familiar music on its own, independent of the anticipated mood-regulating properties emphasized through our survey of key opinion leaders. While the primary outcome of our research on the efficacy of the DTx in our future proof of concept study will be mitigation of anxiety and agitation, the personalization module is expected to lead to dopaminergic and opioid activity in the reward system *via* auditory-reward network connectivity (76, 85). We envision that this music based DTx will be used and implemented by healthcare professionals or family caregivers. Special attention will be devoted to overcoming tensions that may arise due to onboarding or protocol adherence. This approach to development is expected to yield direct benefits for patients, while reducing caregiver burden and the escalating costs associated with the greying of the world.

## Data Availability

The original contributions presented in the study are included in the article/Supplementary materials, further inquiries can be directed to the corresponding author.
